# The Nerve-Sparing Quality (NSQ) Score: A Novel Intraoperative Scoring System for Assessing Nerve-Sparing Quality During Robot-Assisted Radical Prostatectomy—A Concept and Feasibility Study

**DOI:** 10.3390/jcm15082979

**Published:** 2026-04-14

**Authors:** Jakub Kempisty, Krzysztof Balawender, Oskar Dąbrowski, Karol Burdziak

**Affiliations:** Clinic of Urology and Urological Oncology, Fryderyk Chopin University Clinical Hospital in Rzeszów, ul. Szopena 2, 35-055 Rzeszów, Polanddabrowski.oskar@gmail.com (O.D.); k.burdziak93@gmail.com (K.B.)

**Keywords:** robot-assisted radical prostatectomy, nerve-sparing, surgical quality assessment, intraoperative scoring system, robotic surgery training

## Abstract

**Introduction**: Nerve-sparing (NS) during robot-assisted radical prostatectomy (RARP) plays a critical role in postoperative functional recovery, particularly urinary continence and erectile function. Despite the importance of precise neurovascular bundle (NVB) preservation, intraoperative assessment of NS quality remains largely subjective and lacks standardized evaluation tools. The aim of this study was to develop and preliminarily evaluate a structured intraoperative scoring system designed specifically for assessing NS quality during RARP. **Methods**: A novel 10-point intraoperative NS scoring system (NSQ Score) based on five domains was developed: dissection plane, bleeding control, bundle manipulation, continuity of dissection, and symmetry. Each parameter was rated on a 0–2 scale. Thirty robot-assisted radical prostatectomy (RARP) procedures performed in 2024 were randomly selected from a prospectively maintained institutional surgical video archive. Cases were not pre-filtered based on tumor stage, surgical difficulty, or intraoperative complexity. High-definition video recordings of the nerve-sparing phase were anonymized and independently evaluated by three experienced observers blinded to patient outcomes and to each other’s assessments. Inter-rater agreement was analyzed using weighted Cohen’s kappa statistics with quadratic weights, complemented by exact and near-agreement proportions. Cluster bootstrap resampling was applied to account for bilateral observations. **Results**: A total of 48 evaluable observations were analyzed. The overall inter-rater agreement demonstrated a weighted kappa of 0.41 (95% CI 0.36–0.48), indicating fair-to-moderate agreement among reviewers. Exact agreement occurred in 43% of observations, while near-agreement (allowing one ordinal level difference) reached 98%. Among individual parameters, symmetry demonstrated the highest reliability with substantial agreement (κ = 0.70; 95% CI 0.58–0.81). Other domains showed fair agreement, including intraoperative bleeding (κ = 0.36), continuity of dissection (κ = 0.39), bundle manipulation (κ = 0.34), and dissection plane (κ = 0.27). Agreement levels were comparable between left- and right-sided dissections. **Conclusions**: We propose a novel structured intraoperative scoring system for evaluating nerve-sparing quality during RARP. The scale is simple, procedure-specific, and feasible for structured postoperative or video-based assessment. Preliminary results demonstrate fair-to-moderate inter-rater reliability with very high near-agreement, supporting the feasibility of this tool for clinical use. The proposed scoring system may facilitate standardized training, objective performance assessment, and future studies correlating intraoperative NS quality with functional outcomes.

## 1. Introduction

Nerve-sparing (NS) during RARP profoundly influences postoperative continence and erectile function, key determinants of quality of life after prostate cancer surgery [1,2,3]. Since Walsh’s anatomical description of NS radical prostatectomy [4], refinements have aimed to balance oncologic control and neurovascular bundle (NVB) preservation. Robotic systems improved dexterity and 3D visualization, enabling more precise dissection near NVBs [5]. RARP now predominates globally because of ergonomic and visual advantages over open or laparoscopic surgery [6]. Nevertheless, outcomes remain heterogeneous. Large prospective studies such as LAPPRO have demonstrated variability in continence and potency recovery, with subsequent analyses also confirming substantial inter-surgeon heterogeneity in functional and oncological outcomes [7,8]. These differences are largely technique-dependent, especially regarding NS dissection quality [9,10,11]. Despite the availability of high-definition recording and advanced robotic optics, NS assessment remains subjective. Current methods rely on surgeon impression, postoperative function, or surrogate oncologic endpoints. GEARS (Global Evaluative Assessment of Robotic Skills) evaluates generic robotic competencies such as depth perception and dexterity [12]. OSATS (Objective Structured Assessment of Technical Skills) applies global rating scales and checklists across surgical disciplines [13]. PACE (Prostatectomy Assessment and Competency Evaluation) is a prostatectomy-specific instrument focusing on discrete procedural steps [14]. However, most currently available surgical assessment tools evaluate general robotic or laparoscopic skills and are not specifically designed to assess the anatomical precision and technical nuances of nerve-sparing dissection during RARP. Consequently, they provide limited insight into the quality of neurovascular bundle preservation, which is directly associated with postoperative functional outcomes such as urinary continence and erectile function [15].

Consequently, there is a pressing need for a practical, reproducible, and easy-to-apply scoring system tailored specifically to NS in RARP. A concise, reproducible, and NS-specific scoring system could:

Provide structured feedback;

Enable objective training and benchmarking;

Support institutional auditing;

Facilitate AI-assisted surgical evaluation [16].

To address this gap, we developed the Nerve-Sparing Quality (NSQ) Score, a structured intraoperative scoring system designed to objectively evaluate the quality of nerve-sparing during robot-assisted radical prostatectomy. The NSQ Score is a 10-point scoring system based on five domains: dissection plane, bleeding control, bundle manipulation, continuity of dissection, and symmetry. The aim of this study was to develop the NSQ Score and to preliminarily evaluate its feasibility and inter-rater reliability.

## 2. Materials and Methods

### 2.1. Development of the Scoring System

The scale was designed after an extensive review of the literature on intraoperative assessment tools (GEARS, OSATS, PACE) and functional outcome studies [12,13,14,15]. The five domains were identified through a structured review of existing surgical assessment tools and literature addressing functional outcomes after radical prostatectomy, followed by expert consensus among experienced robotic prostatectomy surgeons involved in the development of the scoring framework:

**Tissue Plane**—adherence to the correct anatomical dissection plane, as defined by visible landmarks (Denonvilliers’ fascia, prostatic capsule, periprostatic fascia). Deviations from the correct plane are associated with increased risk of nerve injury and positive margins.

**Bleeding Control**—ability to minimize bleeding in the operative field. Hemostasis is crucial for visibility and for reducing collateral damage caused by cautery near NVBs.

**Nerve Manipulation**—degree of traction, grasping, or stretching applied to NVBs. Gentle handling and minimal traction are key technical principles in nerve-sparing dissection and represent observable components of surgical performance suitable for structured assessment [17].

**Continuity of Dissection**—fluency and consistency of the surgical dissection. Interrupted, fragmented dissection may increase risk of fascial tearing and nerve trauma.

**Symmetry**—comparison between left and right NS dissection.

Scoring definitions were standardized (Table 1). In order to ensure reproducibility of scoring, each side of the prostate was assessed independently for the four technical domains: tissue plane, bleeding control, nerve manipulation, and continuity of dissection. Each domain was graded on a 0–2 scale, resulting in a unilateral score ranging from 0 to 8.

For bilateral nerve-sparing procedures, scores obtained for the left and right sides were averaged to obtain a mean technical dissection score. Subsequently, a global symmetry parameter (0–2 points) was added to the averaged score to reflect the consistency between both sides of the dissection.

The final composite score therefore ranged from 0 to 10.

In unilateral nerve-sparing procedures, only the operated side was assessed and the symmetry parameter was automatically assigned a score of 0, ensuring that the final score accurately reflected the actual extent and quality of nerve preservation.

**Table 1:** Nerve-sparing technique scoring system.

Parameter	0 Points	1 Point	2 Points
Continuity of dissection	Interrupted, chaotic	Moderate continuity	Smooth, uninterrupted
Intraoperative bleeding	Massive, obscuring field	Moderate, controlled	Minimal or none
Dissection plane	Outside anatomical layer	Partially correct	Fully anatomical
Bundle manipulation	Grasping, stretching and/or extensive monopolar or bipolar cauterization close to the bundle	Light touching/tension and/or limited or indirect cautery near the neurovascular bundle	None or very delicate manipulation, only pinpoint coagulation
Symmetry	Significant difference/unilateral	Minor differences	Both sides comparable

Example applications

**Example 1:** Bilateral NS with smooth, continuous dissection, minimal bleeding, no nerve traction, and symmetric technique. Score: 10/10.

**Example 2:** Unilateral NS with partial deviation from correct plane, moderate bleeding controlled promptly, occasional nerve stretching, and fragmented dissection. Symmetry = 0. Total score: 4/10.

**Example 3:** Bilateral NS with smooth right-sided dissection but left-sided irregularity, more bleeding, and occasional nerve grasping. Total score: 6/10.

These examples illustrate the discriminatory ability of the tool to distinguish high- versus low-quality NS.

### 2.2. Case Selection and Video Review Process

Thirty robot-assisted radical prostatectomy procedures performed in 2024 were selected from a prospectively maintained institutional database at a tertiary academic center. Cases were randomly chosen to include both bilateral and unilateral nerve-sparing (NS) procedures. All surgeries were video-recorded in high definition, anonymized, and edited to include only the NS portion of the operation. The videos were then reviewed in iterative sessions by three independent observers experienced in robotic surgery. All reviewers were experienced urologists actively participating in robot-assisted radical prostatectomy, with established experience in robotic prostate cancer surgery and regular involvement in surgical video assessment and training activities. Reviewers were blinded to patient demographics, postoperative outcomes, and to each other’s evaluations. Prior to independent scoring, calibration sessions were conducted to ensure consistent interpretation of the five assessed domains. Each reviewer subsequently evaluated all videos independently, scoring each of the five parameters according to the proposed scale. The average time required per assessment was also recorded for analysis.

All video analyses were performed using high-definition surgical recordings, and scores were recorded in a standardized spreadsheet template for subsequent statistical analysis.

### 2.3. Statistical Analysis

Descriptive statistics for the rating categories were presented as frequencies and percentages for each category within parameters and across raters. Ninety-five percent confidence intervals for these proportions were estimated using the Wilson score interval method.

Inter-rater agreement was assessed using the average pairwise Cohen’s weighted kappa statistic with quadratic weights as the primary measure. This approach accounts for the ordinal nature of the ratings (3-level ordinal scale) by penalizing larger disagreements more severely than minor ones, providing a chance-corrected index of agreement suitable for clinical evaluations involving multiple raters. For three raters, the average of all pairwise weighted kappas was computed to summarize overall concordance, a method recommended for extending kappa to multiple observers while maintaining interpretability. Supplementary measures included the proportion of exact agreement (where all raters assigned identical scores) and the proportion of any agreement (allowing a tolerance of one ordinal level, capturing near-consensus). These proportions offer unadjusted insights into raw and tolerant concordance, complementing the kappa by highlighting patterns of absolute and proximate alignment.

The study design incorporated repeated measures, as some patients underwent bilateral procedures (yielding two observations per patient: left and right sides), while others were unilateral (one observation). To address potential within-patient dependence in bilateral cases, cluster bootstrap resampling was employed, with resampling conducted at the patient level (unique ID) to preserve correlations between sides. This clustering ensures unbiased variance estimation in the presence of non-independent observations. Analyses were stratified to evaluate agreement overall (averaged across all parameters), per clinical parameter (Intraoperative Bleeding, Continuity of Dissection, Bundle Manipulation, Dissection Plane, and Symmetry), and by laterality (left side, right side, and combined). Stratification allowed for detailed examination of agreement variations across domains and anatomical sides.

Point estimates for kappa and agreement proportions were calculated directly from the observed data. In addition, 95% confidence intervals (CIs) were derived using bias-corrected and accelerated (BCa) bootstrap methods with 1000 replications, providing robust, non-parametric inference that accommodates the ordinal data and clustered structure. In cases of perfect agreement (proportion = 1.0) for supplementary measures in non-overall strata, exact binomial CIs were applied as a conservative approximation, ignoring clustering to yield interpretable bounds despite zero variance. *p*-values tested the null hypothesis of no agreement beyond chance (kappa = 0) and were approximated conservatively based on underlying pairwise comparisons, with values below 0.001 reported as such.

Agreement levels were interpreted using established thresholds for kappa: values below 0 indicate poor agreement, 0.00–0.20 slight, 0.21–0.40 fair, 0.41–0.60 moderate, 0.61–0.80 substantial, and 0.81–1.00 almost perfect. These benchmarks facilitate clinical contextualization, though interpretation also considered the supplementary agreement proportions for a comprehensive reliability assessment.

### 2.4. Characteristic of the Statistical Tool

Analyses were conducted using the R Statistical language (version 4.5.2; R Core Team, 2025) on Windows 11 Pro 64 bit (build 26100), using the packages lpSolve (version 5.6.23), ggalluvial (version 0.12.5), boot (version 1.3.32), irr (version 0.84.1), report (version 0.6.2), gtsummary (version 2.4.0), ggplot2 (version 4.0.1), dplyr (version 1.1.4) and tidyr (version 1.3.1).

## 3. Results

### 3.1. Descriptive Analysis of Rating Distributions Across Clinical Parameters and Individual Raters

The study cohort consisted of video recordings derived from RARP procedures focused on neurovascular bundle dissection in a cohort of 30 patients. Among these, 18 patients underwent bilateral procedures, contributing recordings from both left and right sides and generating 36 observations, whereas 12 patients had unilateral nerve-sparing procedures (comprising 5 left-sided and 7 right-sided cases), adding 12 observations for a total of 48 evaluable records. The assessed clinical parameters included intraoperative bleeding, continuity of dissection, bundle manipulation, dissection plane, and symmetry. Detailed frequency distributions of these ratings across raters are presented in Table 2, illustrating a predominance of better outcomes (e.g., minimal bleeding and smooth dissection in over 45% of cases across raters), with notable consistency in moderate to optimal categories for most parameters.

### 3.2. Comprehensive Evaluation of Inter-Rater Agreement Metrics Stratified by Clinical Parameters and Anatomical Laterality

The inter-rater agreement analysis, as summarized in Table 3, indicates moderate overall concordance across all parameters, with an average weighted kappa of 0.41 (95% CI: 0.36–0.48), reflecting fair to moderate chance-adjusted agreement among the three raters. The exact agreement proportion was 0.43 (95% CI: 0.37–0.49), denoting identical ratings in 43% of observations, while the any agreement proportion (with one-level tolerance) reached 0.98 (95% CI: 0.97–0.99).

**Figure 1:**
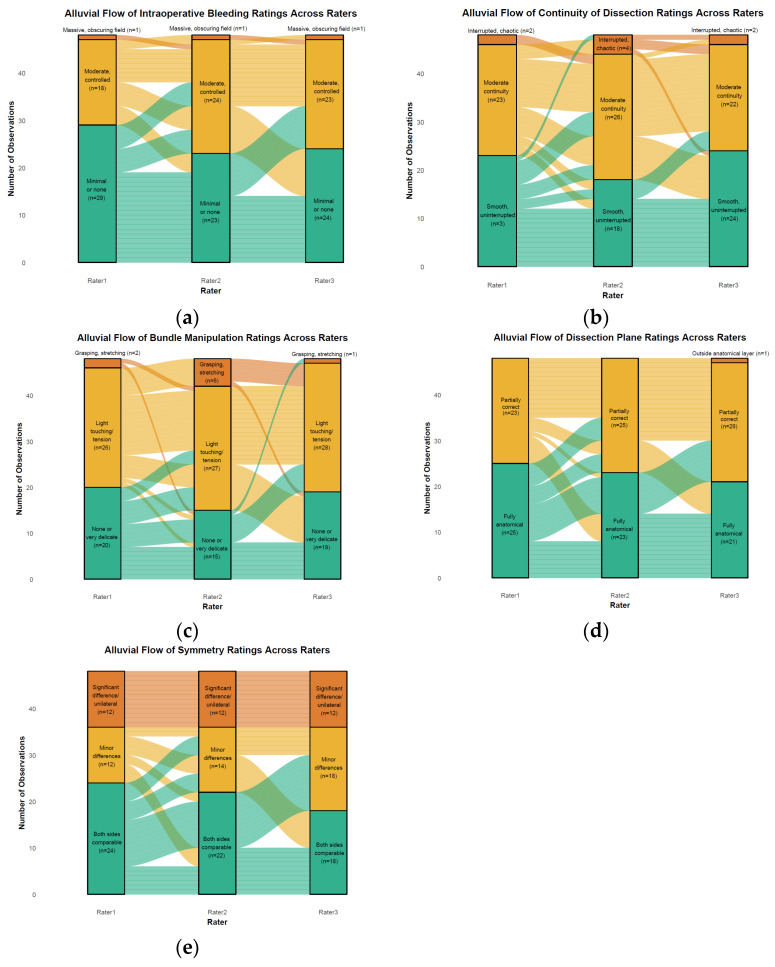
Alluvial diagrams depicting transitions in ratings across three independent raters for each evaluated parameter: (**a**) intraoperative bleeding; (**b**) continuity of dissection; (**c**) bundle manipulation; (**d**) dissection plane; (**e**) symmetry.

Transitions in characteristics between raters are visualized using alluvial diagrams (Figure 1).

Laterality stratification showed similar agreement levels, with weighted kappas of 0.40 (95% CI: 0.31–0.49) for left-sided and 0.37 (95% CI: 0.29–0.47) for right-sided observations. Overlapping confidence intervals confirm no significant differences between sides. Exact and any agreement proportions were also consistent, with any agreement nearing 1.00. Thus, laterality does not significantly affect concordance, though a modest trend of lower kappa estimates on the right side emerged in specific subgroups: intraoperative bleeding (right: 0.31, 95% CI: 0.09–0.50; left: 0.38, 95% CI: 0.10–0.57), bundle manipulation (right: 0.25, 95% CI: 0.04–0.48; left: 0.34, 95% CI: 0.16–0.56), and dissection plane (right: 0.18, 95% CI: 0.04–0.36; left: 0.26, 95% CI: 0.00–0.49). Numerically lower agreement values were observed for right-sided assessments; however, these differences were not statistically significant due to overlapping confidence intervals. Agreement differed markedly by parameter: symmetry yielded the highest kappa of 0.70 (95% CI: 0.58–0.81), rated as substantial, while intraoperative bleeding (0.36; 95% CI: 0.23–0.49), continuity of dissection (0.39; 95% CI: 0.23–0.55), bundle manipulation (0.34; 95% CI: 0.22–0.45), and dissection plane (0.27; 95% CI: 0.13–0.40) displayed fair agreement. Non-overlapping confidence intervals between symmetry and the others confirm significantly superior agreement for symmetry, whereas overlaps among the fair-agreement parameters indicate no significant inter-parameter differences. Within-parameter laterality subgroups mirrored this, with no significant left-right disparities (overlapping intervals), though right-sided dissection plane and bundle manipulation exhibited numerically lower kappas (0.18 and 0.25 vs. left-sided 0.26 and 0.34), hinting at minor variations.

These results highlight parameter-driven effects on agreement, with symmetry’s objectivity fostering stronger concordance, contrasted by fair levels in dynamic parameters like bleeding and manipulation. Laterality exerts negligible influence, affirming scale consistency across sides.

## 4. Discussion

The present study introduces the Nerve-Sparing Quality (NSQ) Score, a structured intraoperative scoring system designed to evaluate the quality of nerve-sparing dissection during robot-assisted radical prostatectomy. The proposed tool is based on five key domains that reflect essential technical aspects of neurovascular bundle preservation: dissection plane, bleeding control, bundle manipulation, continuity of dissection, and symmetry. The development of standardized tools for evaluating surgical technique has become increasingly important as robotic surgery continues to expand and procedural complexity increases [15,16,17,18].

The main finding of this study is that the NSQ Score demonstrated fair-to-moderate inter-rater agreement overall, with particularly high reliability for the symmetry parameter and very high levels of near-consensus between raters. Inter-rater agreement analysis showed an overall weighted kappa of 0.41, corresponding to fair-to-moderate agreement among experienced robotic surgeons. While the proportion of near-agreement was high, the exact agreement rate of 43% indicates that variability between raters remains present. Therefore, the reliability of the NSQ Score should be interpreted as moderate and consistent with observations reported for other structured surgical assessment tools. Comparable agreement levels have been reported for other structured assessment tools developed to evaluate surgical skills and robotic performance [12,14].

Interestingly, agreement varied across the individual parameters of the score. The symmetry domain demonstrated the highest inter-rater reliability, with a weighted kappa of 0.70 corresponding to substantial agreement. This finding likely reflects the relatively objective nature of this parameter, as visual comparison between both sides of the dissection can be performed more consistently than evaluation of more nuanced surgical maneuvers. In contrast, domains such as dissection plane, bundle manipulation, and bleeding control demonstrated lower but still acceptable levels of agreement. These parameters require interpretation of subtle intraoperative movements and tissue interactions, which may naturally introduce variability between observers. Nevertheless, the consistently high near-agreement proportions suggest that the scale remains robust for practical use.

The predominance of higher ratings observed across several parameters may also contribute to the well-known “Kappa paradox”, in which skewed distributions of ratings can lead to moderate kappa values despite high levels of absolute agreement. In such situations, kappa statistics should be interpreted alongside absolute and near-agreement measures to provide a more comprehensive understanding of inter-rater reliability.

Another relevant observation is that laterality did not significantly influence agreement levels. Similar kappa values were observed for both left- and right-sided dissections, with overlapping confidence intervals across parameters. Although slightly lower agreement estimates were observed in some right-sided subgroups, these differences were small and likely reflect normal variability rather than systematic bias. This supports the internal consistency of the NSQ Score across different anatomical contexts within the same procedure.

The proposed score is specific to NS quality, unlike generic tools such as GEARS or OSATS, or comprehensive but retrospective systems such as PACE (Table 4). GEARS evaluates general robotic skills including depth perception, dexterity, and efficiency, but does not specifically address anatomical aspects of nerve-sparing dissection [12]. OSATS provides a widely used framework for assessing surgical performance but lacks procedure-specific anatomical resolution [13]. PACE, while procedure-specific for radical prostatectomy, was designed primarily for retrospective evaluation of entire procedures rather than focused intraoperative assessment of nerve-sparing quality [14]. In contrast, the NSQ Score offers a simple, procedure-specific tool focused on the most functionally relevant step of radical prostatectomy and may facilitate structured evaluation of nerve-sparing dissection.

Recent advances in AI-assisted surgical assessment and automated video analysis highlight the need for standardized, visually interpretable metrics that enable objective evaluation of surgical performance and support automated feedback systems [19,20,21,22]. The five-domain structure of the NSQ Score may provide a useful framework for such approaches, as structured metrics are essential for translating qualitative surgical actions into quantifiable variables. Similarly, the integration of intraoperative technologies for margin assessment further underscores the importance of real-time evaluation tools capable of improving surgical precision [23].

In addition, contemporary EAU guidelines recommend consideration of nerve-sparing in appropriately selected patients whenever oncologically feasible, further underscoring the clinical relevance of standardized tools for intraoperative assessment of nerve-sparing quality [24].

Validated questionnaires such as IIEF-5 and EPIC-26 remain the gold standards for postoperative functional outcome assessment after prostate cancer surgery [25,26,27]. However, these instruments measure outcomes months after surgery and do not directly evaluate intraoperative technical quality. Establishing correlations between intraoperative nerve-sparing quality and postoperative functional outcomes may therefore represent an important step toward linking surgical performance with patient-centered results. Previous studies examining continence recovery, voiding symptoms, and quality of life after radical prostatectomy have highlighted the importance of meticulous surgical technique and functional preservation strategies [2,27].

Structured intraoperative metrics also facilitate reproducible training and objective evaluation of surgical proficiency in robotic surgery programs [17]. Simulation-based robotic curricula and virtual-reality training systems have been shown to improve surgical skill acquisition and enhance feedback mechanisms during training [28,29,30]. Emerging educational frameworks integrating artificial intelligence, data-driven analytics, and competency-based progression further underscore the need for standardized intraoperative metrics that can support objective feedback and longitudinal skill development [31]. Within this context, the five-domain NSQ Score framework may serve as a practical target for structured evaluation of nerve-sparing technique during robotic surgery training programs.

Incorporating objective intraoperative scoring systems into quality assurance frameworks may also support credentialing and benchmarking of robotic surgery performance. Large multi-institutional studies have demonstrated substantial variability in surgical proficiency and outcomes among surgeons performing robot-assisted radical prostatectomy [32]. Standardized evaluation tools may therefore facilitate transparent assessment of surgical quality and support continuous improvement of operative techniques. At the same time, ongoing innovations in radical prostatectomy continue to refine anatomical preservation strategies and perioperative outcomes [33].

Ultimately, a reproducible intraoperative scoring system for nerve-sparing may help bridge the gap between technical surgical performance and functional patient outcomes. Randomized clinical trials have demonstrated comparable oncological efficacy but improved functional recovery following robot-assisted compared with open radical prostatectomy, highlighting the importance of precise nerve preservation techniques [34]. In this context, standardized intraoperative metrics such as the NSQ Score may contribute to better understanding of the relationship between surgical technique and postoperative recovery. Preoperative diagnostic accuracy may also influence surgical planning and nerve-sparing strategies. Studies comparing multiparametric MRI and biopsy findings with final radical prostatectomy pathology highlight the complexity of accurately predicting tumor location and extent prior to surgery [35].

In addition, specific intraoperative technical choices may influence neurovascular bundle preservation. Comparative analyses of cold versus thermal dissection techniques during nerve-sparing RARP suggest that the use of thermal energy may affect nerve integrity and potentially influence postoperative functional recovery [36].

### Study Limitations

This study has some limitations that should be acknowledged. First, a degree of subjectivity remains inherent to the assessment, particularly in evaluating the nuances of nerve manipulation during dissection. Second, the quality of recorded videos and camera angles may influence scoring accuracy and interobserver reliability. This work represents an initial developmental phase of the proposed scoring system. Third, the current validation is limited to a single institution and a relatively small sample size, which may restrict the generalizability of the findings. Finally, while the scale is conceptually linked to functional recovery, this correlation remains theoretical until confirmed by ongoing prospective, multicenter trials.

## 5. Conclusions

We propose the Nerve-Sparing Quality (NSQ) Score, a novel structured intraoperative scoring system designed to evaluate the quality of nerve-sparing during robot-assisted radical prostatectomy. Compared with existing instruments such as GEARS, OSATS, and PACE, the proposed scale offers several advantages, including simplicity, procedure specificity, and feasibility for real-time or immediate postoperative assessment.

The NSQ Score provides a practical framework for the structured evaluation of neurovascular bundle preservation, enabling objective assessment of key technical aspects of nerve-sparing dissection. By focusing on five intuitive domains—dissection plane, bleeding control, bundle manipulation, continuity of dissection, and symmetry—the scale allows surgeons to evaluate nerve-sparing quality both during the procedure and through delayed video-based analysis.

The primary goal of this work was to develop a simple and reproducible tool that facilitates the assessment of nerve-sparing quality, which may support surgical education, structured feedback, and continuous improvement of operative technique. The ability to evaluate nerve-sparing performance in a standardized manner may facilitate learning of atraumatic neurovascular bundle preservation and contribute to improving the overall quality of nerve-sparing during robotic prostatectomy.

In this feasibility study, evaluation of neurovascular bundle dissection demonstrated fair-to-moderate inter-rater agreement, with particularly strong agreement for the symmetry parameter and consistently high near-agreement across all assessed domains. These findings support the practical applicability and reproducibility of the NSQ Score in clinical settings.

By bridging the gap between subjective intraoperative impressions and structured evaluation of surgical technique, the NSQ Score may represent a valuable tool for surgical training, quality monitoring, and future research exploring the relationship between intraoperative nerve-sparing quality and postoperative functional outcomes. The NSQ Score may also provide a standardized framework for evaluating nerve-sparing quality in future clinical studies and robotic surgery training programs, facilitating objective comparison of surgical techniques across institutions.

## Figures and Tables

**Table 2:** Descriptive statistics of ratings by parameter and rater (N = 48).

Characteristic	Rater 1	Rater 2	Rater 3
**Intraoperative Bleeding**			
Massive, obscuring field	1 (2.08%; CI: 0.11–12%)	1 (2.08%; CI: 0.11–12%)	1 (2.08%; CI: 0.11–12%)
Moderate, controlled	18 (37.50%; CI: 24–53%)	24 (50.00%; CI: 36–64%)	23 (47.92%; CI: 34–63%)
Minimal or none	29 (60.42%; CI: 45–74%)	23 (47.92%; CI: 34–63%)	24 (50.00%; CI: 36–64%)
**Continuity of Dissection**			
Interrupted, chaotic	2 (4.17%; CI: 0.72–15%)	4 (8.33%; CI: 2.7–21%)	2 (4.17%; CI: 0.72–15%)
Moderate continuity	23 (47.92%; CI: 34–63%)	26 (54.17%; CI: 39–68%)	22 (45.83%; CI: 32–61%)
Smooth, uninterrupted	23 (47.92%; CI: 34–63%)	18 (37.50%; CI: 24–53%)	24 (50.00%; CI: 36–64%)
**Bundle Manipulation**			
Grasping, stretching	2 (4.17%; CI: 0.72–15%)	6 (12.50%; CI: 5.2–26%)	1 (2.08%; CI: 0.11–12%)
Light touching/tension	26 (54.17%; CI: 39–68%)	27 (56.25%; CI: 41–70%)	28 (58.33%; CI: 43–72%)
None or very delicate	20 (41.67%; CI: 28–57%)	15 (31.25%; CI: 19–46%)	19 (39.58%; CI: 26–55%)
**Dissection Plane**			
Outside anatomical layer	-	-	1 (2.08%; CI: 0.11–12%)
Partially correct	23 (47.92%; CI: 34–63%)	25 (52.08%; CI: 37–66%)	26 (54.17%; CI: 39–68%)
Fully anatomical	25 (52.08%; CI: 37–66%)	23 (47.92%; CI: 34–63%)	21 (43.75%; CI: 30–59%)
**Symmetry**			
Significant difference/unilateral	12 (25.00%; CI: 14–40%)	12 (25.00%; CI: 14–40%)	12 (25.00%; CI: 14–40%)
Minor differences	12 (25.00%; CI: 14–40%)	14 (29.17%; CI: 17–44%)	18 (37.50%; CI: 24–53%)
Both sides comparable	24 (50.00%; CI: 36–64%)	22 (45.83%; CI: 32–61%)	18 (37.50%; CI: 24–53%)

Note: Statistics are reported in the form of frequencies (percentages with 95% CI). Abbreviation: CI = Confidence Interval. Dashes (-) indicate zero occurrences for the specified category. Percentages and confidence intervals are based on the Wilson method.

**Table 3:** Inter-rater agreement metrics for clinical parameters stratified by laterality.

Stratification	Unique Patients	Observations	Weighted Kappa (95% CI)	Exact Agreement Proportion (95% CI)	Any Agreement Proportion (95% CI)
**Overall (All Parameters)**	30	48	0.41 (0.36, 0.48)	0.43 (0.37, 0.49)	0.98 (0.97, 0.99)
Overall (Left Only)	23	23	0.40 (0.31, 0.49)	0.41 (0.33, 0.48)	0.99 (0.98, 1.00)
Overall (Right Only)	25	25	0.37 (0.29, 0.47)	0.45 (0.39, 0.52)	0.98 (0.96, 1.00)
**Intraoperative Bleeding (All)**	30	48	0.36 (0.23, 0.49)	0.44 (0.31, 0.55)	1.00 (0.93, 1.00)
Intraoperative Bleeding (Left)	23	23	0.38 (0.10, 0.57)	0.43 (0.27, 0.59)	1.00 (0.85, 1.00)
Intraoperative Bleeding (Right)	25	25	0.31 (0.09, 0.50)	0.44 (0.31, 0.60)	1.00 (0.86, 1.00)
**Continuity of Dissection (All)**	30	48	0.39 (0.23, 0.55)	0.46 (0.33, 0.57)	0.96 (0.91, 1.00)
Continuity of Dissection (Left)	23	23	0.35 (0.17, 0.53)	0.43 (0.28, 0.60)	0.96 (0.92, 1.00)
Continuity of Dissection (Right)	25	25	0.38 (0.16, 0.58)	0.48 (0.35, 0.64)	0.96 (0.93, 1.00)
**Bundle Manipulation (All)**	30	48	0.34 (0.22, 0.45)	0.42 (0.28, 0.54)	0.96 (0.91, 1.00)
Bundle Manipulation (Left)	23	23	0.34 (0.16, 0.56)	0.39 (0.25, 0.54)	1.00 (0.85, 1.00)
Bundle Manipulation (Right)	25	25	0.25 (0.04, 0.48)	0.44 (0.29, 0.60)	0.92 (0.83, 1.00)
**Dissection Plane (All)**	30	48	0.27 (0.13, 0.40)	0.42 (0.31, 0.52)	1.00 (0.93, 1.00)
Dissection Plane (Left)	23	23	0.26 (0.00, 0.49)	0.39 (0.23, 0.54)	1.00 (0.85, 1.00)
Dissection Plane (Right)	25	25	0.18 (0.04, 0.36)	0.44 (0.29, 0.60)	1.00 (0.86, 1.00)
**Symmetry (All)**	30	48	0.70 (0.58, 0.81)	0.42 (0.27, 0.56)	1.00 (0.93, 1.00)
Symmetry (Left)	23	23	0.67 (0.46, 0.80)	0.39 (0.23, 0.54)	1.00 (0.85, 1.00)
Symmetry (Right)	25	25	0.73 (0.55, 0.83)	0.44 (0.29, 0.60)	1.00 (0.86, 1.00)

Note: Weighted kappa represents average pairwise Cohen’s kappa with quadratic weights. Exact agreement is the proportion of observations with identical ratings across all raters. Any agreement allows a tolerance of one ordinal level. Confidence intervals (CIs) are 95% bias-corrected and accelerated bootstrap intervals (1000 replications, clustered by patient); for perfect agreement proportions (1.00), exact binomial CIs were used conservatively. All kappa coefficients were significantly different from zero; however, interpretation focuses primarily on the magnitude of agreement rather than statistical significance. Bold rows indicate summaries across all sides for each parameter or overall.

**Table 4:** Comparison of existing evaluation tools.

Tool	Focus	Strengths	Limitations	NS-Specific?	Real-Time Feasible?
**GEARS**	Generic robotic skills	Validated, structured	Not procedure-specific	No	Yes
**OSATS**	Generic technical skills	Widely used	Lacks anatomy nuance	No	Yes
**PACE**	Radical prostatectomy	Procedure-specific, validated	Retrospective, time-consuming	Partly	No
**Proposed NS Score**	Nerve-sparing quality	Simple, fast, reproducible	Needs validation	Yes	Yes

## Data Availability

The original contributions presented in this study are included in the article. Further inquiries can be directed to the corresponding author.
